# Incidence, clinical features, and implications on outcomes of neonatal late-onset sepsis with concurrent infectious focus

**DOI:** 10.1186/s12879-017-2574-7

**Published:** 2017-07-03

**Authors:** I-Hsyuan Wu, Ming-Horng Tsai, Mei-Yin Lai, Lee-Fen Hsu, Ming-Chou Chiang, Reyin Lien, Ren-Huei Fu, Hsuan-Rong Huang, Shih-Ming Chu, Jen-Fu Hsu

**Affiliations:** 1 Division of Pediatric Neonatology, Department of Pediatrics, Chang Gung Memorial Hospital, No. 5, Fu-Shin Rd., Kwei-Shan, Taoyuan, Linkou Chang Gung Memorial Hospital, Taoyuan, Taiwan, Republic of China; 2 Division of Neonatology and Pediatric Hematology/Oncology, Department of Pediatrics, Chang Gung Memorial Hospital, Yunlin, Taiwan; 3 College of Medicine, Chang Gung University, Taoyuan, Taiwan; 4 Department of Respiratory Care, Chang Gung University of Science and Technology, Chia-Yi, Taiwan

**Keywords:** Bacteremia, Neonates, Catheter-related bloodstream infection, Late-onset sepsis, Risk factor

## Abstract

**Background:**

Neonatal bloodstream infection (BSI) is the most important cause of morbidity and mortality in the neonatal intensive care unit (NICU). Although most neonatal BSIs are primary bacteremia, some are associated with a focus of infection. This distinction is not well characterized.

**Methods:**

All patients with neonatal late-onset sepsis (LOS) between January 2006 and December 2013 were enrolled. LOS was categorized as a BSI with a concurrent focus of infection if LOS occurred before or within 24 h after the diagnosis of a specific infectious entity, and as “primary bacteremia” if no concurrent focus of infection was identified. Data concerning demographics, hospital course, microbiology, and outcomes were compared via univariate and multivariate analyses.

**Results:**

Of 948 episodes of neonatal LOS, 781 (82.4%) were primary bacteremia, whereas 167 (17.6%) were associated with a known focus of infection, including meningitis (*n* = 51, 5.4%), ventilator-associated pneumonia (VAP) (*n* = 36, 3.8%), catheter-related bloodstream infections (*n* = 57, 6.0%), and necrotizing enterocolitis (NEC) (*n* = 21, 2.2%). The majority of NEC-associated BSIs were caused by gram-negative bacilli (85.7%). Group B streptococcus accounted for nearly one-third of all meningitis cases (29.4%). Although sepsis-attributable mortality was comparable between primary bacteremia and neonatal BSIs with a focus of infection, neonatal BSIs with meningitis, VAP, and NEC had significantly higher rates of infectious complications. The independent risk factors of sepsis-attributable mortality were infectious complications (Odds ratio [OR] 6.98; 95% confidence interval [CI] 3.64–13.39, *P* < 0.001); history of one or more than one previous episode(s) of BSI (OR 2.40 and 7.40; 95% CI 1.21–4.74 and 3.70–14.78, *P* = 0.012 and <0.001, respectively); and underlying secondary pulmonary hypertension in neonates (OR 4.77; 95% CI 1.91–11.96, *P* = 0.001).

**Conclusions:**

A considerable proportion of neonatal LOS can be associated with known infectious foci in the NICU. The microbiologic etiology of neonatal LOS with a concurrent focus of infection is significantly different from that of primary bacteremia. Neonatal BSIs with concurrent meningitis, VAP, or NEC are significantly more likely to have infectious complications. This association independently leads to sepsis-attributable mortality.

## Background

Bloodstream infection (BSI) is the most common nosocomial infection in the neonatal intensive care unit (NICU) [1, 2], and accounts for the most important cause of morbidity and mortality after these neonates have survived the perinatal insults and complications of extreme prematurity [3, 4]. Neonates with certain underlying chronic comorbidities are more likely to have severe infection or die after BSI [5, 6]. Factors predisposing to morbidity or mortality after neonatal BSI include prolonged use of a central catheter and/or ventilation, recurrent nosocomial infections, and possibly, immunosuppression caused by the use of broad-spectrum antibiotics [3–5]. Similarly, concurrent neonatal BSIs with infectious complications are often more severe, and sometimes may require surgical intervention [7, 8].

Our recent study has demonstrated that concurrent meningitis and ventilator-associated pneumonia were independent predictors of treatment failure in neonatal BSI [6]. Bizzarro et al. also showed that infants with necrotizing enterocolitis (NEC)-associated BSI are significantly more likely to die than are those with post-NEC BSI and NEC without BSI [9]. An episode of BSI with concurrent infectious focus may impose greater therapeutic challenge, and adequate infectious source control plays an important role in optimizing treatment response. There have been several studies in adults that investigated BSI with concurrent skin and soft tissue infection, bacteriuria, gastroenteritis, or pneumonia [10–14], but the epidemiology and outcomes of neonatal BSIs with a concurrent infectious entity are poorly characterized. Therefore, we conducted a retrospective cohort study to determine the risk factors for, specific microbiology of, and clinical implications of neonatal BSI with a concurrent focus of infection.

## Methods

### Study setting, subjects and ethics

The Chang Gung Memorial Hospital (CGMH) is a tertiary-level medical center in a university-affiliated teaching hospital in northern Taiwan. The CGMH’s NICU has three units that include a total of 49 beds equipped with mechanical ventilators, and 28 beds in special-care nurseries. All babies under 34–35 weeks gestation or birth weight < 2 kg or >5 kg, and those with any clinical signs of respiratory distress or cardiovascular, gastrointestinal, or neurological problems requiring surgical or intensive treatment were eligible for admission to this NICU. We identified all episodes of culture-proven BSI in the NICU of CGMH between 1 January 2006 and 31 December 2013. All BSIs identified were “late-onset”, defined as a positive blood culture occurring at >72 h of life [1, 4, 15]. This study was approved by the institutional review board of Chang Gung Memorial Hospital, with a waiver of informed consent because all patient records and information were anonymized and de-identified prior to analysis.

### Data retrieval and study database

For more than ten years, the NICU at CGMH has had an electronic database maintained by a full-time nurse specialist dedicated to following neonates from birth (or admission if the neonate was transferred from another hospital) until discharge or death. The NICU database contains information on basic demographic data, perinatal insults, and complications of prematurity, a summary of the patient’s hospital course, chronic comorbidities, and discharge diagnosis. Utilizing this database, we characterized in detail the course of every episode of BSI, including the clinical manifestations, laboratory results, microbiological data, concurrent focus of infection, treatment, infectious complications, and outcomes. The severity of illness was evaluated at the time of greatest severity during the course of BSI using the neonatal therapeutic intervention scoring system (NTISS) [16].

### Definitions

An episode of BSI was defined according to the presence of clinical sepsis and the identification of pathogens, which included any bacteria isolated from at least one blood culture and not consisting of saprophytic skin flora [17]. *Corynebacterium* spp., *Propionibacterium* spp., *Penicillium* spp., and *Diphtheroids* spp. were considered contaminants in blood cultures, and were excluded from consideration. For coagulase-negative staphylococci (CoNS), the diagnosis of BSI required clinical signs of sepsis, and a blood culture positive for CoNS. The indicated treatment for CoNS BSI included intravenous antibacterial therapy for at least 5 days after the date of the blood culture (or until death) [1, 4]. We defined all concurrent infectious foci, including NEC, ventilator associated pneumonia (VAP), central line-associated bloodstream infection (CLABSI), and meningitis according to the U.S. Centers for Disease Control and Prevention’s (CDC’s) National Healthcare Safety Network (NHSN) definitions [8, 18–20]. Only cases with NEC ≥ stage IIA in modified Bell’s criteria (definite NEC) were enrolled, and clinically defined VAP cases were considered [8]. An episode of BSI was defined as “BSI with concurrent infectious focus” if the onset of BSI (defined as the timing of the first positive blood culture was drawn) was accompanied with a specific infectious entity, i.e. meningitis, VAP, or NEC, otherwise, this BSI was categorized as “primary bacteremia”. In this definition, the onset of BSI was always within 24 h (before or after) of the diagnosis of a specific infectious entity.

A patient was said to have an episode of clinical sepsis if the patient had a positive blood culture and was treated with antibiotic therapy for 5 or more days (or was treated for a shorter period if the patient died) and had at least two of the following clinical symptoms of sepsis: fever or hypothermia, hyper- or hypoglycemia, apnea or tachypnea, frequent oxygen desaturation with an increased requirement for ventilator support, bradycardia and/or cyanosis, feeding intolerance, abdominal distension, seizures, decreased motor activity, skin mottling, and hypotension [1, 4]. The microbiological assay system in our hospital is a matrix-assisted laser desorption ionization time-of-flight (MALDI-TOF) system (Bruker’s flagship FLEX series) [21].

The diagnosis of all co-morbidities of prematurity, including respiratory distress syndrome (RDS), intra-ventricular hemorrhage (IVH), broncho-pulmonary dysplasia (BPD), necrotizing enterocolitis (NEC), and peri-ventricular leukomalacia (PVL) was based on the latest updated diagnostic criteria [22]. Congenital anomalies included either documented or undocumented syndromes, chromosomal abnormalities, and genetic or metabolic disorders, but not simple cleft palate or polydactyly. Persistent BSI or fungemia was defined as three or more consecutive positive blood cultures, at least 48 h apart, during a single episode of sepsis [23].

Infectious complications were defined as a new-onset focus of infection, empyema, abscess, venous thrombosis, or vegetation directly related to bacteremia or major organ dysfunction within 1 week after the onset of bacteremia. Empiric antibiotic therapy was considered inappropriate if the treatment regimen did not include at least one antibiotic active in vitro against the infecting microorganisms, administered within 24 h of blood culture collection. For patients who died during hospitalization, the cause of death was recorded according to the clinician’s assessment. Sepsis-attributable mortality was defined as death of a neonate within 3 days after the onset of sepsis or death from of infectious complications or clinically progressive deterioration following the onset of BSI.

### Statistical analysis

Descriptive data all episodes of neonatal BSI were expressed as mean and standard deviation (SD) or median and IQR (interquartile range), where appropriate, for continuous data and absolute number and percentage for dichotomous data. Infecting organisms responsible for BSI in infants with infectious entities associated and primary BSI were compared via χ^2^ analysis and presented as unadjusted OR with 95% CI. If an infant had multiple episodes of sepsis, each episode was considered as an independent event.

In univariate analyses, continuous data were analyzed using the Kruskal-Wallis and Mann-Whitney U tests, where appropriate. Dichotomous data were analyzed using χ^2^ test and Fisher exact test, where appropriate. A *P* value <0.05 was considered statistically significant. BSI-attributable mortality was assessed as the dependent variable in a multivariate logistic regression model. This model incorporated variables identified in a univariate regression model with a *P* value of <0.10. Estimates were expressed as odds ratios (ORs) with 95% confidence intervals (CIs). All statistical analyses were performed using SPSS version 21.0 (SPSS, Chicago, IL, USA).

## Results

A total of 948 episodes of BSI were identified in 732 infants during this study period, of which 781 (82.4%) were primary bacteremia and 167 (17.6%) had a specific focus of infection. The incidence rate of neonatal BSI during this study period was 3.71 per 1000 neonate-hospital days. Among those with concurrent infection, 51 (45%) BSIs had meningitis, 36 (23%) were VAP, 21 (23%) were NEC, and 57 (28%) were CLABSI. Other uncommon foci of infection included osteomyelitis (2), septic arthritis (2), upper limbs cellulitis (1), and urinary tract infection (1). Among these 167 BSIs with a focus of infection, five (3.0%) had more than two infectious foci. The microbiology of BSIs with concurrent infection was distinctively different from that of primary bacteremia (Table 1). The majority of NEC-associated BSIs were caused by gram-negative bacilli (85.7%) and group B streptococcus accounted for nearly one-third of all meningitis cases (29.4%). More than half cases of VAP- and CLABSI-associated BSIs were attributable to gram-negative bacilli (61.1%) and gram-positive pathogens (64.9%), respectively.

**Table 1:** Pathogens causing a total of 948 episodes of primary bloodstream infection (BSI) and 123 episodes of BSI with specific infectious focus in the neonatal intensive care unit

	Neonatal BSIs with specific infectious focus (total *n* = 167)					Primary bacteramia (total *n* = 781)
	Meningitis (total *n* = 51)	Ventilator associated pneumonia (*n* = 36)	CLABSI (*n* = 57)	Necrotizing enterocolitis (*n* = 21)	Others^c^ (*n* = 7)	
Gram-positive organism						
Coagulase-negative *Staphylococcus*	4 (7.8)	0 (0)	25 (43.9)	3 (14.3)	1 (14.3)	332 (42.5)
*Staphylococcus aureus*	9 (17.6)	9 (25.0)	11 (19.3)	0 (0)	4 (57.1)	89 (11.4)
*Enterococcus* species	2 (3.9)	1 (2.8)	1 (1.8)	0 (0)	0 (0)	22 (2.8)
*Group-B streptococcus*	15 (29.4)	1 (2.8)	0 (0)	0 (0)	0 (0)	10 (1.3)
*Streptococcus pneumoniae*	0 (0)	1 (2.8)	0 (0)	0 (0)	0 (0)	2 (0.2)
*Viridan Streptococcus*	0 (0)	0 (0)	0 (0)	0 (0)	0 (0)	2 (0.2)
Gram-negative organism						
*Klebsiella pneumoniae*	5 (9.8)	6 (16.7)	5 (8.8)	3 (14.3)	0 (0)	72 (9.2)
*Klebsiella oxytoca*	0 (0)	1 (2.8)	1 (1.8)	0 (0)	0 (0)	22 (2.8)
*Escherichia coli*	7 (13.7)	3 (8.3)	2 (3.5)	8 (38.1)	1 (14.3)	59 (7.5)
*Enterobacter cloacae*	1 (2.0)	1 (2.8)	1 (1.8)	0 (0)	0 (0)	21 (2.7)
*Enterobacter aerogenes*	0 (0)	1 (2.8)	1 (1.8)	1 (4.8)	0 (0)	13 (1.7)
*Pseudomonas aeruginosa*	2 (3.9)	2 (5.6)	1 (1.8)	1 (4.8)	0 (0)	10 (1.3)
*Acinetobacter baumannii*	1 (2.0)	3 (8.3)	1 (1.8)	1 (4.8)	0 (0)	36 (4.6)
*Serratia marcescens*	2 (3.9)	1 (2.8)	2 (3.5)	2 (9.5)	0 (0)	6 (0.8)
*Others* ^*a*^	2 (3.9)	1 (2.8)	1 (1.8)	0 (0)	0 (0)	8 (1.3)
Fungus						
*Candida albicans*	1 (2.0)	0 (0)	4 (7.0)	2 (5.6)	0 (0)	18 (2.3)
*Candida parapsilosis*	0 (0)	0 (0)	1 (1.8)	0 (0)	0 (0)	13 (1.7)
Other *Candida* spp.	0 (0)	0 (0)	0 (0)	0 (0)	0 (0)	11 (1.4)
Polymicrobial microorganisms^b^	0 (0)	3 (8.3)	0 (0)	2 (9.5)	1 (14.3)	35 (4.5)

*CLABSI* central line-associated bloodstream infection

^a^Including *Citrobacter freundii* (3), *Stenotrophomonas maltophilia* (3), *Hafnia alvei* (2), *Neisseria meningitidis* (2), *Chryseobacterium meningoseptium* (1) and *Flavobacterium* (1)

^b^Indicating two or more microorganisms were recovered from the same blood culture set

^c^Including osteomyelitis (2), septic arthritis (2), upper limbs cellulitis (2), and urinary tract infection (1)

Demography, hospital course, underlying chronic comorbidities, and treatment for all neonatal BSIs with concurrent infection were evaluated comparing with primary BSIs (Table 2). Neonates with BSIs concurrent with meningitis had a significantly higher birth weight and later gestational age than neonates with primary bacteremia, whereas neonates with BSIs concurrent with VAP had a significantly lower birth weight and gestational age than neonates with primary bacteremia. Neonatal BSIs with concurrent NEC and neonatal BSIs with concurrent VAP tended to occur earlier and later than primary bacteremia, respectively. VAP associated BSIs were more likely to occur in neonates with low Apgar score (≤ 7) at five minutes and in those with perinatal asphyxia.

**Table 2:** Episodes of neonatal bloodstream infections stratified according to primary bacteremia and bacteremia with various foci of infection

Characteristics	Neonatal BSIs with specific focus of infection (total *n* = 167)					Primary bacteremia (*n* = 781)
	Meningitis (*n* = 51)	VAP (*n* = 36)	CLABSI (*n* = 57)	NEC (*n* = 21)	Others^b^ (*n* = 7)	
Demographic characteristics						
Birth body weight (g), median (IQR)	2375 (1405–2905)**	960 (820–1600)**	1267.5 (911–2680)	1480 (1065–2040)*	2330 (1600–2700)*	1240 (880–1880)
Gestational age (weeks), median (IQR)	35.0 (30.0–38.0)**	27.0 (25.0–31.0)**	29.5 (27.0–37.0)	29.0 (27.0–34.0)	37.0 (32–38)**	30.0 (27.0–34.0)
Male sex, n (%)	30 (58.8)	21 (58.3)	29 (50.9)	10 (47.6)	5 (71.4)	405 (51.9)
Onset of BSI (day), median (IQR)	33.5 (17.8–60.3)	30 (20–58)*	25 (17–45.5)	19 (12–43)**	42 (11–73)	28 (17.0–53.0)
Perinatal history						
Low Apgar score at 5 min (≦ 7)	13 (25.5)**	22 (61.1)**	26 (45.6)	5 (23.8)**	2 (28.6)**	351 (44.9)
Perinatal asphyxia	6 (11.8)	13 (36.1)**	4 (7.0)	2 (9.5)	0 (0)	62 (7.9)
Underlying chronic conditions^a^						
Congenital anomalies	2 (3.9)	2 (5.6)	4 (7.0)	0 (0)	2 (28.6)	55 (7.0)
Neurological comorbidities	23 (45.1)**	5 (13.9)	3 (5.3)	1 (4.8)	1 (14.3)	91 (11.7)
Complicated congenital heart disease	0 (0)	0 (0)	3 (5.3)	0 (0)	0 (0)	32 (4.1)
Acyanotic CHD with heart failure	0 (0)	1 (2.8)	3 (5.3)	0 (0)	0 (0)	17 (2.2)
Bronchopulmonary dysplasia	9 (17.6)	23 (63.9)**	15 (26.3)	6 (28.6)	2 (28.6)	243 (31.1)
Pulmonary hypertension	1 (2.0)	1 (2.8)	3 (5.3)	0 (0)	0 (0)	23 (2.9)
Gastrointestinal pathology	0 (0)	1 (2.8)	4 (7.0)	3 (14.3)	0 (0)	54 (6.9)
Renal	1 (2.0)	1 (2.8)	2 (3.5)	1 (4.8)	0 (0)	25 (3.2)
Cholestasis^b^	4 (7.8)	9 (25.0)	13 (22.8)	6 (28.6)	4 (57.1)**	147 (18.8)
Clinical septic symptoms						
Fever (temperature > 38 °C)	33 (64.7)	8 (22.2)	26 (45.6)	6 (28.6)	4 (57.1)	319 (40.8)
Apnea ± bradycardia and/or cyanosis	31 (60.8)	32 (88.9)	40 (70.2)	16 (76.2)	5 (71.4)	528 (67.6)
Abdominal distension ± feeding intolerance	31 (60.8)	27 (75.0)*	27 (47.4)	21 (100)**	5 (71.4)*	457 (58.5)
Tachycardia	15 (29.4)	5 (13.9)	13 (22.8)	6 (28.6)	1 (14.3)	177 (22.7)
Hyper- or hypoglycemia	14 (27.5)	14 (38.9)	11 (19.3)	8 (38.1)	1 (14.3)	206 (26.4)
Septic shock	8 (15.7)	11 (30.6)*	6 (10.5)	9 (42.9)**	2 (28.6)	126 (16.1)
Disseminated intravascular coagulopathy	8 (15.7)*	5 (13.9)	3 (5.3)	5 (23.8)*	1 (14.3)	62 (7.9)
Laboratory parameter						
Leukopenia (WBC count <4000/uL)	12 (23.5)	6 (16.7)	5 (8.8)	9 (42.9)**	3 (42.9)**	127 (16.3)
Leukocytosis (WBC count >20,000/uL)	14 (27.5)	14 (38.9)*	13 (22.8)	5 (23.8)	4 (57.1)**	217 (27.8)
WBC shift to left (immature WBC ≥ 20%)	10 (19.6)	7 (19.4)	3 (5.3)	7 (33.3)**	1 (14.3)	89 (11.4)
Anemia (hemoglobin <11.0 mg/dL)	22 (43.1)	23 (63.9)**	26 (45.6)	13 (61.9)**	2 (28.6)	291 (37.3)
Thrombocytopenia (platelet <80,000/uL)	16 (31.4)	21 (58.3)**	19 (33.3)	11 (52.4)**	1 (14.3)	267 (34.2)
Metabolic acidosis	12 (23.5)	15 (41.7)**	6 (11.8)	10 (47.7)**	1 (14.3)	139 (17.8)
Prolonged PT and/or aPTT	12 (23.5)	11 (30.5)	12 (21.1)	11 (52.4)**	1 (14.3)	196 (25.1)
Sequences of BSI during hospitalization						
1st episode	41 (80.4)	27 (75.0)	42 (73.7)	19 (90.5)	4 (57.1)	584 (74.8)
2nd episode	7 (13.7)	6 (16.7)	12 (21.1)	1 (4.8)	2 (28.6)	126 (16.1)
3rd (or greater) episode	3 (5.9)	3 (8.3)	3 (5.3)	1 (4.8)	1 (14.3)	71 (9.1)

All data were expressed as number (percentage %), unless indicated otherwise

*NTISS* neonatal therapeutic intervention scoring system, *IQR* interquartile range, *SD* standard deviation, *BSI* bloodstream infection, *NSD* natural vaginal delivery, *C/S* cesarean section

*P < 0.05; **P < 0.001; *P* values are the comparisons between BSIs with specific infectious focus and primary bacteremia

^a^Indicating the presence of chronic conditions or comorbidities at onset of bloodstream infection, and some patients had >1 underlying chronic conditions. Each patient with an underlying chronic condition is compared with those without that specific condition

^b^Indicating direct bilirubin ≥1.5 mg/dL or more than 50% of the total bilirubin

The underlying chronic comorbidities were not significantly different between primary bacteremia and BSIs with a concurrent focus of infection, except that VAP-associated BSIs and meningitis were more likely to occur in neonates with BPD and neurological comorbidities, respectively. In addition to GBS meningitis, other gram-positive-cocci–associated meningitis occurred mostly in neonates with congenital or acquired hydrocephalus as a complication of ventriculoperitonal shunt or extraventricular drainage. All episodes of primary bacteremia, as well as neonatal BSIs with concurrent meningitis, VAP, or CLABSI were evenly distributed as first episode of LOS or recurrent episode of LOS; however, there were only two cases of BSI with concurrent NEC occurring as a recurrent episode of LOS.

The clinical presentations of neonatal BSI with meningitis and CLABSIs were mostly comparable to those of primary bacteremia; however, NEC-associated and VAP-associated BSIs had more severe clinical symptoms, including more septic shock, disseminated intravascular coagulopathy, thrombocytopenia, anemia, and metabolic acidosis. Appropriate antibiotics were administrated in 72.2% of cases of primary bacteremia within 24 h of onset, which was similar to the rate in cases of neonatal BSIs with concurrent VAP, NEC, and CLABSIs.

Neonatal BSIs with concurrent VAP and NEC had significantly greater severity of illness, judged by NTISS scores (Table 3). Neonates with VAP-associated BSIs were more likely to require prolonged invasive intubation (≥ 7 days) and to experience respiratory failure under a high-frequency oscillatory ventilator. Neonatal BSIs with concurrent meningitis, VAP, and NEC had significantly higher rates of infectious complications than did primary bacteremia. The sepsis-attributable mortality rates were comparable between primary bacteremia and BSIs with a concurrent focus of infection.

**Table 3:** Treatment and outcomes of neonatal bloodstream infections among the three CRP groups

	Neonatal BSIs with specific infectious focus (total *n* = 167)					Primary bacteremia (*n* = 781)
	Meningitis (*n* = 51)	VAP (*n* = 36)	CLABSI (*n* = 57)	NEC (*n* = 21)	Others^#^ (*n* = 7)	
NTISS score, (mean ± SD)	15.4 ± 5.1	19.8 ± 4.0**	16.8 ± 4.0	19.0 ± 6.5**	14.7 ± 6.1	16.9 ± 4.5
Ventilator requirement						
Prolonged invasive intubation (≥ 7 days)	14 (27.5)	22 (61.1)**	19 (33.3)	8 (38.0)	2 (28.6)	287 (36.7)
High frequency oscillatory ventilator	0 (0)	14 (38.9)**	3 (5.3)	4 (19.0)*	0 (0)	71 (9.1)
Inadequate antibiotic treatment within the first 24 h	10 (19.6)*	8 (22.2)	17 (29.8)	7 (33.3)	4 (57.1)**	217 (27.8)
Outcomes						
Infectious complications	18 (35.3)**	10 (27.8)**	6 (10.5)	5 (23.8)**	0 (0)	68 (8.7)
Sepsis attributable mortality	5 (9.8)	4 (11.1)	3 (5.3)	3 (14.3)	1 (14.3)	53 (6.8)

The *P* values are the comparisons between neonatal BSIs with infectious focus and primary bacteremia

*P < 0.05

**P < 0.001

Table 4 shows univariate comparisons of neonatal BSIs with sepsis-attributable mortality versus those without. None of neonatal BSIs with concurrent meningitis, VAP, or NEC was associated with significantly higher rates of sepsis-attributable mortality. Underlying secondary pulmonary hypertension (due to severe BPD), neurological comorbidities, presence of infectious complications, and history of one or more previous episode(s) of neonatal BSI were significantly associated with a higher risk of sepsis-attributable mortality. In multivariate logistic regression, the presence of infectious complications (Odds ratio [OR] 6.98; 95% confidence interval [CI] 3.64–13.39, *P* < 0.001), history of one or more previous episode(s) of BSI (OR 2.40 and 7.40; 95% CI 1.21–4.74 and 3.70–14.78, *P* = 0.012 and <0.001, respectively), and underlying secondary pulmonary hypertension (OR 4.77; 95% CI 1.91–11.96, *P* = 0.001) were independent predictors of sepsis-attributable mortality.

**Table 4:** Risk factors for sepsis-attributable mortality in neonatal bloodstream infections with and without a focus of infection

Variables	Univariate analysis		Multivariate logistic regression analysis	
	Unadjusted OR (95% CI)	*P* value	Adjusted OR (95% CI)	*P* value
Gestational age (weeks)	0.95 (0.90–1.01)	0.126	-	-
Inappropriate antibiotics	1.64 (1.01–2.73)	0.048	1.05 (0.57–1.94)	0.872
Presence of infectious complications	6.19 (3.61–10.62)	< 0.001	6.98 (3.64–13.39)	< 0.001
Sequence of bloodstream infections				
1st episode	1 (reference)		1 (reference)	
2nd episode	2.90 (1.57–5.41)	0.001	2.40 (1.21–4.74)	0.012
3rd (or greater) episode	8.53 (4.63–15.71)	< 0.001	7.40 (3.70–14.78)	< 0.001
Underlying pulmonary hypertension	8.11 (3.58–18.35)	< 0.001	4.77 (1.91–11.96)	0.001
Underlying neurological comorbidities	2.80 (1.59–4.93)	< 0.001	1.89 (0.99–3.61)	0.053
BSIs with and without infectious focus				
Primary bacteremia	1 (reference)		-	-
With concurrent meningitis	1.05 (0.34–3.21)	0.937	-	-
With concurrent ventilator-associated pneumonia	1.27 (0.38–4.20)	0.699	-	-
With necrotizing enterocolitis	2.84 (0.64–12.57)	0.169	-	-
With concurrent CLABSIs	1.07 (0.34–3.75)	0.918	-	-

*CLABSI* central line-associated bloodstream infection, *OR* odds ratio, *95% CI* 95% confidence interval

## Discussion

In this study, we found that the majority of neonatal late-onset BSIs were primary bacteremia, and only 17.6% were associated with a specific focus of infection. To our knowledge, this is the first investigation of neonatal BSIs with concurrent onset of nosocomial infections. Not surprisingly, the microbiologic profile of neonatal BSI concurrent with a specific focus of infection was significantly different from that of primary bacteremia. In contrast to previous studies documenting that patients with NEC-associated BSI and GBS meningitis have higher mortality rates than do those with bacteremia [9, 24], we found that a concurrent focus of infection did not directly contribute to case mortality. Instead, a concurrent focus of infection was associated with a significantly higher rate of infectious complications, and the associations finally lead to sepsis attributable mortality.

The overall incidence of culture-positive meningitis, VAP, NEC, and the distribution of organisms in this cohort was similar to other studies [9, 24–26]. Although neonates with concurrent VAP and NEC seemed more severely ill, the insignificant influence of neonatal meningitis, VAP, and NEC on sepsis-attributable mortality can be explained partially by the small sample size. Our previous studies showed that neonates with fungal BSI and multidrug-resistant gram-negative bacteremia have a significantly higher mortality rate [1, 5]. Because these pathogens usually caused primary bacteremia in this study, the sepsis-attributable mortality was comparable between primary bacteremia and bacteremia with concurrent infectious focus. Furthermore, our aggressive treatment strategies for neonatal NEC, as well as meningitis and VAP, could possibly reduce the mortality rate of these disease entities [1, 2, 5, 6].

Although our study extended over a relatively long period of time, there were very few variations in practice and all collections of data showed similar results (data not shown). The sepsis-attributable mortality rates in our cohort, both in the primary bacteremia group and in the group with BSI with a concurrent focus of infection, were much lower than those reported in other studies [9, 27]. For neonatal BSIs with meningitis, the case fatality rate of our cohort was comparable to that reported in some recent studies [24, 28, 29]. Previous studies have shown worse outcomes in preterm or small for gestational age infants [28–30], but this was not documented in our study. It is therefore likely that the presence of underlying chronic comorbidities contributed to mortality [1, 6], both in primary bacteremia and neonatal BSIs with a concurrent focus of infection.

A recent study found that infants with blood-cerebrospinal fluid (CSF) concordance were more likely to have indicators related to increased severity of illness [26]. Because neonatal meningitis is inevitably associated with significantly higher rates of mortality and morbidity [6, 7, 31], the relatively lower sepsis-attributable mortality of neonatal BSI with meningitis in this cohort highlighted the importance of aggressive treatment. Based on this result, the clinicians should consider treating the primary bacteremia and controlling the infectious focus at the same time once a focus is identified. The antibiotic regimens and treatment duration of neonatal bacteremia with concurrent meningitis or NEC are supposed to be adjusted to the presence of infectious complications.

CLABSI is an important source of neonatal BSI. In this study, we applied strict diagnostic criteria for CLABSI [18]. CLABSI was diagnosed only in neonates from whom the catheter was removed at the time of or soon after the onset of the episode of BSI, and in whom culture of the catheter tip was positive for the same organism that was cultured from the blood. Cultures are imperfect, and sometimes the catheter was removed after empiric antibiotics had been administrated. Therefore, we may have inadvertently missed patients who, in fact, had an unidentified focus of infection in the catheter. Furthermore, some CLABSIs were treated with the catheter in situ, which led to an underestimate of the true incidence of neonatal BSI due to CLABSIs. Nonetheless, it is clear that in almost all demographics, clinical characteristics, treatment, and outcomes neonatal BSIs due to CLABSIs were comparable to those details in primary bacteremia.

The presence of certain underlying chronic comorbidities may increase the risk of secondary bacteremia. In our cohort, only neurological comorbidities and BPD predisposed neonates to higher rates of meningitis and VAP, respectively. The frequency of secondary bacteremia in neonates with nosocomial infections other than BSIs was low [32, 33], but it should be considered when there are clinical symptoms lasting more than 48 h or when signs or symptoms of sepsis have reemerged.

The sources of neonatal BSIs were identified in only 17.6% episodes in this cohort. It has been proven that the sources of neonatal BSIs are most commonly from epidermis colonization and gram-negative bacilli penetration of bowel mucosa, for gram-positive cocci and gram-negative bacilli, respectively [34, 35]. Because not all infected catheters were removed for bacterial culture at onset of BSIs, the percentage of neonatal BSIs with concurrent CLABSI in this cohort may be underestimated.

The microbiological assay system is MALDI-TOF in our institute, which has the advantage of rapid identification and reliable microbiological results [36, 37]. However, the disadvantage of this method is the lacking tests of antibiotic resistances; therefore, additional antibiotic susceptibility testing was required in our institute.

This study has some limitations. This was a retrospective, single-center cohort study, which inevitably restricts its generalizability compared with that of a prospective, multicenter study. We applied the definition of single positive blood culture for CoNS with clinical symptoms, which possibly resulted in an over-estimate of the burden of CoNS-related bacteremia. Subgroup analyses were limited by sample size, and some episodes of BSIs with a focus of infection may have been treated with antibiotics while awaiting blood culture results. The delay in identifying a specific focus may have resulted in sterilization of the CSF, sputum, or catheter tips. Blood cultures were ordered as clinically indicated, and the pathogens might not have been identified in some patients with infectious sources. Further, the identification of concurrent infectious was based on clinical judgment, which can be subjective.

## Conclusions

Our data suggest that neonatal BSIs with a concurrent focus of infection is not rare, and is associated with increased severity of illness and a higher rate of infectious complications. At the initial presentation of neonatal BSI, systemic evaluation should be performed to identify a possible source of infection after blood cultures are obtained. In the support and recovery phase of neonatal BSIs with a concurrent focus of infection, especially in cases with VAP, meningitis, and NEC, efforts to limit the risk of infectious complications and prolonged hospitalization are necessary, because infectious complications are independently associated with in-hospital mortality.

## Abbreviations

BPD: Bronchopulmonary dysplasia
BSI: Bloodstream infection
CDC: Centers for disease control and prevention
CI: Confidence interval
CoNS: Coagulase negative staphylococcus
CRBSI: Catheter-related bloodstream infection
CRP: C-reactive protein
CVC: Central venous catheter
ESBL: Extended-spectrum β-lactamase
GBS: Group B streptococcus
GPC: Gram-positive cocci
IQR: Interquartile range
MALDI-TOF: Matrix-assisted laser desorption ionization time-of-flight
NEC: Necrotizing enterocolitis
NICU: Neonatal intensive care unit
NTISS: Neonatal therapeutic intervention scoring system
OR: Odds ratio
PVL: Periventricular leukomalacia
RDS: Respiratory distress syndrome
TPN: Total parenteral nutrition
VAP: Ventilator associated pneumonia

## References

[1] Tsai MH, Hsu JF, Chu SM, Lien R, Huang HR, Chiang MC (2014). Incidence, clinical characteristics, and risk factors of adverse outcome in neonates with late-onset sepsis. Pediatr Infect Dis J.

[2] Tsai MH, Wu IH, Lee CW, Chu SM, Lien R, Huang HR (2016). Neonatal gram-negative bacillary late-onset sepsis: a case-control-control study on a prospectively collected database of 5233 admissions. Am J Infect Control.

[3] Tsai MH, Lien R, Wang JW, Huang HR, Chiang CC, Chu SM (2009). Complication rates with percutaneously central venous catheters inserted at femoral and non-femoral sites in very low birth weight infants. Pediatr Infect Dis J.

[4] Tsai MH, Chu SM, Lee CW, Hsu JF, Huang HR, Chiang MC (2014). Recurrent late-onset sepsis in the neonatal intensive care unit: incidence, clinical characteristics, and risk factors. Clin Microbiol Infect.

[5] Tsai MH, Chu SM, Hsu JF, Lien R, Huang HR, Chiang MC (2014). Risk factors and outcomes for multidrug-resistant gram-negative bacteremia in the NICU. Pediatrics.

[6] Hsu JF, Chu SM, Huang YC, Lien R, Huang HR, Lee CW, Chiang MC, Fu RH, Tsai MH. Predictors of clinical and microbiological treatment failure in neonatal bloodstream infections. Clin Microbiol Infect 2015;21:e482.e9–482.e17. 10.1016/j.cmi.2015.01.009 25749002

[7] Chu SM, Hsu JF, Lee CW, Lien R, Huang HR, Chiang MC, Fu RH, Tsai MH (2014). Neurological complications after neonatal bacteremia: the clinical characteristics, risk factors, and outcomes. PLoS One.

[8] Chu SM, Yang MC, Hsiao HF, Hsu JF, Lien R, Chiang MC, Fu RH, Huang HR, Hsu KH, Tsai MH (2015). One-week versus 2-day ventilator circuit change in neonates with prolonged ventilation: cost-effectiveness and impact on ventilator-associated pneumonia. Infect Control Hosp Epidemiol.

[9] Bizzarro MJ, Ehrenkranz RA, Gallagher PG (2014). Concurrent bloodstream infection in infants with necrotizing enterocolitis. J Pediatr.

[10] Tattevin P, Schwartz BS, Graber CJ, Volinski J, Bhukhen A, Mai TT (2012). Concurrent epidemics of skin and soft tissue infection and bloodstream infection due to community-associated methicillin-resistant Staphylococcus Aureus. Clin Infect Dis.

[11] Yoon YK, Lee J, Ryu SY, Chang HH, Choi WS, Yoon JH (2013). Clinical significance of multidrug-resistant Acinetobacter Baumannii isolated from central venous catheter tip cultures in patients without concomitant bacteremia. Scand J Infect Dis.

[12] Schreiber MP, Chan CM, Shorr AF (2011). Bacteremia in Staphylococcus Aureus pneumonia: outcomes and epidemiology. J Crit Care.

[13] Choi SH, Lee SO, Choi JP, Lim SK, Chung JW, Choi SH (2009). The clinical significance of concurrent Staphylococcus Aureus bacteriuria in patients with S. Aureus bacteremia. J Inf Secur.

[14] Puerta-Fernandez S, Miralles-Linares F, Sanchez-Simonet MV, Bernal-Lopez MR, Gomez-Huelgas R (2013). Raoultella planticola bacteraemia secondary to gastroenteritis. Clin Microbiol Infect.

[15] Verstraete EH, Blot K, Mahieu L, Vogelaers D, Blot S (2015). Prediction models for neonatal health care-associated sepsis: a meta-analysis. Pediatrics.

[16] Gray JE, Richardson DK, McCormick MC, Workman-Daniels K, Goldmann DA (1992). Neonatal therapeutic intervention scoring system: a therapy-based severity-of-illness index. Pediatrics.

[17] Opota O, Croxatto A, Prod’hom G, Greub G (2015). Blood culture-based diagnosis of bacteraemia: state of the art. Clin Microbiol Infect.

[18] Mehta Y, Jaggi N, Rosenthal VD, Kavathekar M, Sakle A, Munshi N (2016). Device-associated infection rates in 20 cites of India, data summary for 2004-2014: findings of the international nosocomial infection control consortium. Infect Control Hosp Epidemiol.

[19] Tsai MH, Chu SM, Hsu JF, Lien R, Huang HR, Chiang MC (2015). Breakthrough bacteremia in the neonatal intensive care unit: incidence, risk factors, and attributable mortality. Am J Infect Control.

[20] Al-Mousa HH, Omar AA, Rosenthal VD, Salama MF, Aly NY, El-Dossoky Noweir M (2016). Device-associated infection rates, bacterial resistance, length of stay, and mortality in Kuwait: international nosocomial infection consortium findings. Am J Infect Control.

[21] Rodriguez-Sánchez B, Sánchez-Carrillo C, Ruiz A, Marin M, Cercenado E, Rodrigez-Créixems M, Bouza E (2014). Direct identification of pathogens from positive blood cultures using matrix-assisted laser desorption-ionization time-of-flight mass spectrometry. Clin Microbiol Infect.

[22] Gleason CA, Devaskar SU (2011). Avery’s disease of the newborn.

[23] Hsu JF, Chu SM, Lee CW, Yang PH, Lien R, Chiang MC (2015). Incidence, clinical characteristics and attributable mortality of persistent bloodstream infection in the neonatal intensive care unit. PLoS One.

[24] Joubrel C, Tazi A, Six A, Dmytruk N, Touak G, Bidet P (2015). Group B streptococcus neonatal invasive infection, France 2007-2012. Clin Microbiol Infect.

[25] Stoll BJ, Hansen NI, Bell EF, Shankaran S, Laptook AR, Walsh MC (2010). Neonatal outcomes of extremely preterm infants from the NICHD neonatal research network. Pediatrics.

[26] Beam KS, Laughon MM, Hornik CP, Cohen-Wolkowiez M, Clark RH, Benjamin DK (2014). Predictors of positive cerebrospinal fluid cultures in infants with bacteremia. Pediatr Infect Dis J.

[27] Schwab F, Zibell R, Piening B, Geffers C, Gastmeier P (2015). Mortality due to bloodstream infections and necrotizing enterocolitis in very low birth weight infants. Pediatr Infect Dis J.

[28] Okike IO, Johnson AP, Henderson KL, Blackburn RM, Muller-Pebody B, Ladhani SN (2014). Incidence, etiology, and outcome of bacterial meningitis in infants aged < 90 days in the United Kingdom and Republic of Ireland: prospective, enhanced, national population-based surveillance. Clin Infect Dis.

[29] Gaschignard J, Levy C, Romain O, Cohen R, Bingen E, Aujard Y, Boileau P (2011). Neonatal bacterial meningitis: 444 cases in 7 years. Pediatr Infect Dis J.

[30] Escobar GJ, Puopolo KM, Wi S, Turk BJ, Kuzniewicz MW, Walsh EM (2014). Stratification of risk of early-onset sepsis in newborn ≧ 34 weeks’ gestation. Pediatrics.

[31] Shane AL, Hansen NI, Stoll BJ, Bell EF, Sánchez PJ, Shankaran S (2012). Methicillin-resistant and susceptible Staphylococcus Aureus bacteremia and meningitis in preterm infants. Pediatrics.

[32] Gözmen S, Sükran Gözmen K, Apa H, Aktürk H, Sorguç Y, Bayram N (2014). Secondary bacteremia in rotavirus gastroenteritis. Pediatr Infect Dis J.

[33] Lowenthal A, Livni G, Amir J, Samra Z, Ashkenazi S (2006). Secondary bacteremia after rotavirus gastroenteritis in infancy. Pediatrics.

[34] Niño DF, Sodhi CP, Hackam DJ (2016). Necrotizing enterocolitis: new insights into pathogenesis and mechanisms. Nat Rev Gastroenterol Hepatol.

[35] Meropol SB, Stange KC, Jacobs MR, Weiss JK, Bajaksouzian S, Bonomo RA. Bacterial colonization and antibiotic resistance in a prospective cohort of newborn infants during the first year of life. Open Forum Infect Dis 2016; 3: ofw221. 10.1093/ofid/ofw221 PMC5146758 27957505

[36] Osthoff M, Gurtler N, Bassetti S, Balestra G, Marsch S, Pargger H (2017). Impact of MALDI-TOF-MS-based identification directly from positive blood cultures on patient management: a controlled clinical trial. Clin Microbiol Infect.

[37] Huang AM, Newton D, Kunapuli A, Gandhi TN, Washer LL, Lsip J (2013). Impact of rapid organism identification via matrix-assisted laser desorption/ionization time-of-flight combined with antimicrobial stewardship team intervention in adult patients with bacteremia and candidemia. Clin Infect Dis.

